# Ketogenic Diets: Side Effects, Attitude, and Quality of Life

**DOI:** 10.7759/cureus.20390

**Published:** 2021-12-13

**Authors:** Hani Shalabi, Ahmed Alotaibi, Abdulrahman Alqahtani, Hashim Alattas, Ziyad Alghamdi

**Affiliations:** 1 Department of Internal Medicine, College of Medicine, University of Jeddah, Jeddah, SAU

**Keywords:** behavioral therapy, obesity, high fat diet, low carbohydrate diet, quality of life, ketogenic diet

## Abstract

Introduction: The ketogenic diet has been in use since the 1920s as a therapy for epilepsy. Since the 1960s, it has also become widely known as one of the methods for obesity treatment. Recently, this diet has been promoted as a lifestyle, making it highly controversial in terms of its practicality as a lifestyle diet and its duration without affecting one’s health or quality of life. Hence, this study assessed ketogenic diets from the people’s perspective of side effects, attitude, and quality of life.

Method: This retrospective observational study evaluated people who experienced or still practice a ketogenic diet. Health-related quality of life, the standard four-item set of healthy days core questions, was employed. We distributed the survey as an electronic self-assessment using Google Forms. The data were reviewed and automatically copied into a personal computer, arranged in a data-sheet in Microsoft Excel, and analyzed using Statistical Package for the Social Sciences version 27 (Armonk, NY: IBM Corp.). The data were mainly expressed as numbers and percentages.

Results: A total of 226 subjects who adopted a ketogenic diet were interviewed to explore their diet experience. Females were slightly more than males (52.7% vs. 47.3%), and more than one-half (55.3%) of this study population aged 18-35 years. Obesity accounted for 55.3%, and the majority of the respondents (69.9%) adopted a ketogenic diet for more than one month. Among the most frequently reported symptoms were nausea (mild, 29.2%; moderate, 16.4%; severe nausea, 5.8%), dizziness (mild, 39.8%; moderate, 27.4%; severe, 11.5%), polyuria (72.1% in total), and lethargy (69.7%). Furthermore, 90.3% of them felt happy about adopting a ketogenic diet, and 81.9% would recommend it for anyone who wants to lose weight.

Conclusion: A ketogenic diet was practiced mostly for one to six months, making it a short-term solution to weight loss. The outcomes of the participants approved the efficacy of the ketogenic diet in weight reduction. Different symptoms and side effects occurred with varying intensities, especially in the first few days of adopting this diet. Overall, the ketogenic diet did not affect the quality of life and yielded a very positive overall experience.

## Introduction

Overweight and obesity are health-impairing conditions characterized by abnormal or excessive fat accumulation. They are considered an epidemic that is largely caused by an environment that promotes excessive food intake and discourages physical activity in all ages. In 2008, over 200 million men and nearly 300 million women aged 20 years and over were obese, and 65% of the world’s population lives in countries where being overweight and obese is more detrimental to people than being underweight. Obesity is one of the major risk factors for cardiovascular disease, and along with dyslipidemia, hypertension, and diabetes, it contributes to metabolic syndrome [1]. Obesity is a difficult issue to deal with, but weight loss can be achieved by several solutions focusing mainly on decreasing energy intake and increasing energy consumption; such solutions include exercise and behavioral, pharmacologic, surgical, and device interventions [2].

The ketogenic diet, which is a high-fat, adequate-protein, and low-carbohydrate diet, gained a resurgence of interest during the past decade for the treatment of difficult-to-control seizures in children [3]. Also called a very low-carbohydrate diet, the ketogenic diet has been in use since the 1920s for epilepsy therapy; in some cases, it can completely eliminate the need for medication. From the 1960s onwards, it has become widely known as one of the most common methods for obesity treatment [4].

However, the ketogenic diet is not considered a benign, holistic, or all-natural treatment. As with any serious medical therapy, it may result in complications, although generally less severe and less frequent than those of medication or surgery [5]. Furthermore, most of the studies regarding this diet evaluated pediatric populations with common side effects, including constipation, low-grade acidosis, and hypoglycemia [6]. Cholesterol levels may also increase by approximately 30% [5].

Recently, the ketogenic diet has been promoted as a lifestyle, but it is highly debated in terms of how practical to adopt it as a lifestyle and how long people can continue on such a diet without affecting their health or quality of life. In this study, we aimed to assess behavioral aspects/experiences of ketogenic diet from people’s perspective of side effects, attitude, and quality of life.

## Materials and methods

Study design and period

A retrospective cross-sectional study was conducted among people who experienced or still practice a ketogenic diet. In the preparatory period (four weeks), we composed our study title; conducted a literature review where we collected data about common side effects and the measurement of quality of life; obtained permission from the Bioethics Committee for Scientific and Medical Research at the University of Jeddah; and prepared a questionnaire. Next, we conducted our fieldwork (four weeks), including searching for the target population over social media, designing a Google form for the survey, and testing the form by running few samples to ensure the results appear in the intended structure. After another four weeks, we collected and analyzed our data and finally wrote our report.

Inclusion and exclusion criteria

We included any adult who experienced or still practices a ketogenic diet and can speak Arabic. The study is not directed toward a specific region or country. Those who were below the age of 18 years and who never experienced a ketogenic diet were excluded during the initial assessment questions. They were then directed to a different survey. The sample size was calculated automatically using Google Forms. Out of 359 enrolled individuals, 226 experienced a ketogenic diet, and 133 were excluded.

Data collection tool

We developed an Arabic questionnaire to collect necessary information about ketogenic diet attitudes and quality of life (questionnaire in the Appendices section). Our questionnaire adopted health-related quality of life (HRQOL) measures, the standard four-item set of healthy days core questions developed by the Center for Disease Control and Prevention (CDC HRQOL-4). The CDC HRQOL-4 measures had acceptable test-retest reliability and strong internal validity, which has been used by the CDC and its partners, for tracking population health status and HRQOL measures in states and communities [7]. The questionnaire consists of the following four main parts: (i) sociodemographic data (health condition, weight, and height), (ii) questions assessing the possible side effects, (iii) questions assessing participants’ attitude toward ketogenic diets, and (iv) questions assessing participants’ quality of life.

Data collection technique

We conducted the survey in an electronic self-assessment format using Google Forms. We targeted ketogenic diet groups on social media for over one month through messages and direct contact with group moderators and ensured that only people who practiced the ketogenic diet participated in the survey. One of the first questions in the survey was “have you tried ketogenic diet?” If the answer was no, then the participant was directed to a different questionnaire about diet in general. The data obtained from the survey were reviewed and automatically copied into a personal computer.

Data entry and analysis

Data were arranged as data sheets in Microsoft Excel and analyzed using Statistical Package for the Social Sciences version 27 (Armonk, NY: IBM Corp.). These data were expressed mainly by numbers and percentages. We used a pie chart to describe change in weight, chi-square test to evaluate participants’ perceived happiness, and the mean median and maximum with interquartile range (IQR) to describe the number of days of mental and physical exhaustion.

Ethical considerations

This study was approved by the Bioethics Committee for Scientific and Medical Research at the University of Jeddah (approval no. UJ-REC-007). As a prerequisite for data collection, individual consent was written on the front page of the questionnaire, stating that answering this questionnaire means agreeing to participate in the study. All information was kept confidential and only used for the purpose of scientific research.

## Results

According to the study design, 226 participants who adopted a ketogenic diet were interviewed to explore their ketogenic diet experience and to determine its consequences on their physical and mental health. Characteristics of the study group females were slightly more than males (52.7% vs. 47.3%), and more than one-half of the participants (55.3%) aged 18-35 years. In addition, 55.3% had obesity and 26.1% had morbid obesity (Table 1).

**Table 1:** Characteristics of the respondents (n = 226) BMI: body mass index

Characteristics		Number	Percentage (%)
Gender	Male	107	47.3
	Female	119	52.7
Age categories	<18 years	8	3.5
	18-35 years	125	55.3
	36-55 years	88	38.9
	>55 years	5	2.2
BMI categories	Within normal	28	12.4
	Overweight	73	32.3
	Obesity	66	29.2
	Morbid obesity	59	26.1

Most of the respondents (69.9%) reported that they adopted a ketogenic diet for more than one month. Among them, 12.8% followed it for six to 12 months, and 7.5% for more than one year. Two-thirds (65.5%) of the participants claimed that it was their first time to experience a ketogenic diet, and the second time for 14.6%. Furthermore, 17.3% reported that they experienced it more than thrice.

During the ketogenic diet, more than one-half of the respondents (58.4%) maintained physical exercise. Meanwhile, 54.4% had cheat meals and 42.5% of them had no specific routine. Almost one-half of the participants (48.7%) depended on social media as a source for preparing their ketogenic diet, whereas only 2.2% consulted dietitian (Table 2).

**Table 2:** Practicing a ketogenic diet (n = 226)

Practices during a ketogenic diet			No.	Percentage (%)
Duration	<1 month		68	30.1
	1–<3 months		78	34.5
	3–<6 months		34	15.0
	6–<12 months		29	12.8
	≥12 months		17	7.5
Frequency	Once		148	65.5
	Twice		33	14.6
	3 times		6	2.7
	>3 times		39	17.3
Regular performance of physical exercise	Yes		132	58.4
	No		94	41.6
Cheat meals	No		103	45.6
	Yes	2 cheat meals weekly	4	1.8
		1 cheat meal weekly	23	10.2
		No specific routine	96	42.5
Manner of preparing food	Self-prepared		90	39.8
	Ready-made meals		4	1.8
	Through a dietitian		5	2.2
	Through an experienced friend		12	5.3
	By help of nutrition books		5	2.2
	Through social media		110	48.7

**Figure 1:**
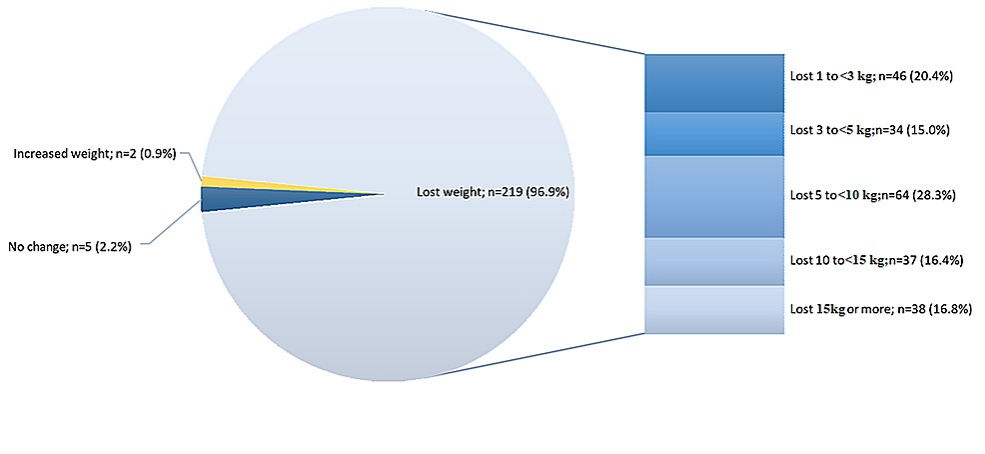
Change in weight as a response to the ketogenic diet

Figure 1 displays the change in weight in response to adopting a ketogenic diet. Majority of the respondents (96.9%) reported that they lost weight, ranging between 1 kg to less than 3 kg (20.4%) and 15 kg or more (16.8%). Only two respondents (0.9%) reported weight gain, and five claimed to have no weight change.

The overall experience of a ketogenic diet was assessed by asking the respondents for any symptoms experienced during the ketogenic diet adoption, particularly in the first three months. Table 3 shows that among the most frequently reported symptoms were nausea (mild, 29.2%; moderate, 16.4%; severe, 5.8%), dizziness (mild, 39.8%; moderate, 27.4%; severe, 11.5%), polyurea (72.1% in total), and sluggishness (69.7%).

**Table 3:** Self-perceived physical symptoms that occurred in the first three months of adopting a ketogenic diet

Symptoms	Mild	Moderate	Severe	Not sure	Not at all
Nausea	66 (29.2)	37 (16.4)	13 (5.8)	13 (5.8)	97 (42.9)
Vomiting	12 (5.3)	6 (2.7)	3 (1.3)	7 (3.1)	198 (87.6)
Dizziness	90 (39.8)	62 (27.4)	26 (11.5)	2 (0.9)	46 (20.4)
Constipation	45 (19.9)	88 (38.9)	23 (10.2)	6 (2.7)	64 (28.3)
Sluggishness	67 (29.6)	62 (27.4)	33 (14.6)	1 (0.4)	63 (27.9)
Hypoglycemia	40 (17.7)	16 (7.1)	11 (4.9)	41 (18.1)	118 (52.2)
Halitosis	52 (23.0)	38 (16.8)	31 (13.7)	24 (10.6)	81 (35.8)
Abdominal pain	43 (19.0)	19 (8.4)	5 (2.2)	14 (6.2)	145 (64.2)
Muscle pain	50 (22.1)	47 (20.8)	16 (7.1)	7 (3.1)	106 (46.9)
Cold or flu	38 (16.8)	13 (5.8)	2 (0.9)	9(4.0)	164 (72.6)
Fever	17 (7.5)	10 (4.4)	1 (0.4)	7 (3.1)	191 (84.5)
Acidosis	4 (1.8)	2 (0.9)	1 (0.4)	51 (22.6)	168 (74.3)
Palpitation	67 (29.6)	29 (12.8)	4 (1.8)	18 (8.0)	108 (47.8)
Polyurea	60 (26.5)	66 (29.2)	37 (16.4)	18 (8.0)	45 (19.9)

**Figure 2:**
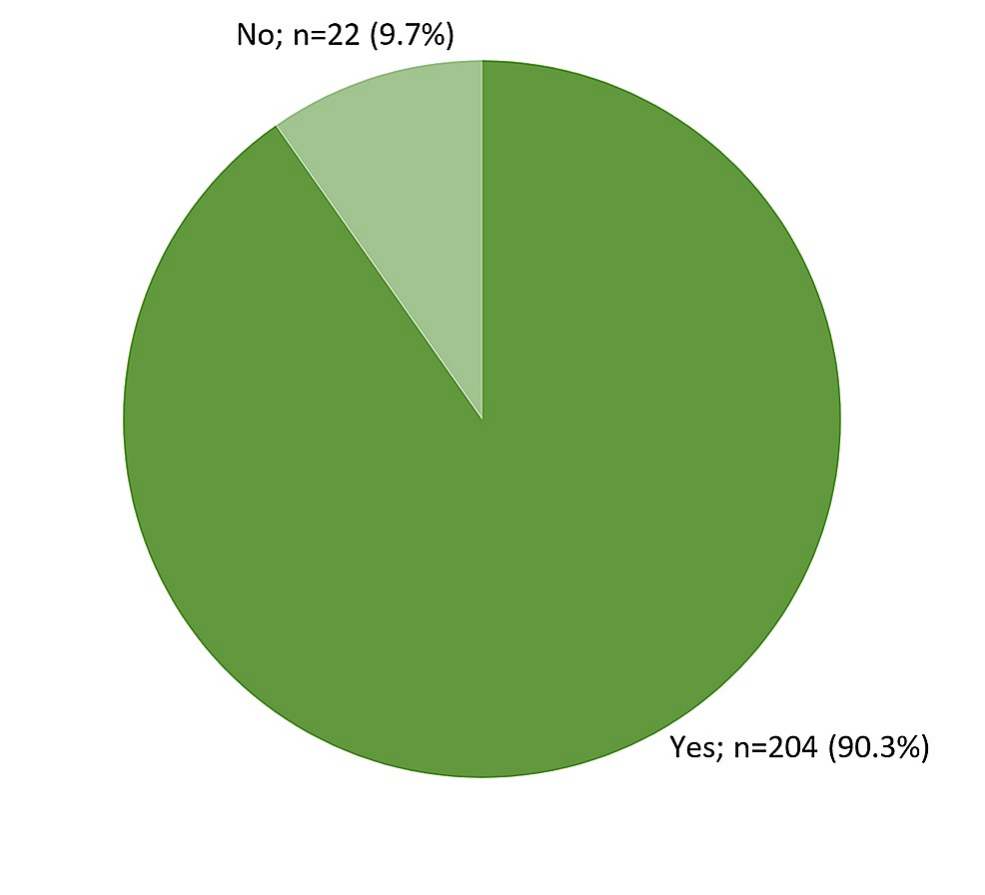
Perceived happiness regarding the ketogenic diet

Table 4 summarizes the overall self-reported perception of the respondents about their physical and mental health in their first month of the ketogenic diet. On average, the respondents reportedly felt poor physical and mental health in two days with an IQR of four days, individually, preventing them from doing usual daily tasks for one day. As shown in Figure 2, most respondents (90.3%) felt happy about adopting a ketogenic diet, and 81.9% of them would recommend this diet to anyone who wants to lose weight.

**Table 4:** Average number of days of perceived overall mental and physical health IQR: interquartile range

Self-reported evaluation of mental and physical health	Median	Minimum	Maximum	IQR
Number of days feeling poor physical health	2	0	30	4
Number of days feeling poor mental health	2	0	30	4
Number of days where poor physical or mental health prevents usual daily tasks	1	0	29	4

Although happiness was felt more in males than in females (93.5% vs. 87.4%), in the age group of 36-55 years (95.5%) than in other age groups, and in those with normal weight (96.4%) than in those with abnormally low or high weight, these differences showed no statistical significance (p > 0.05) (Table 5).

**Table 5:** Perceived happiness about ketogenic diet according to the characteristics of the respondents *Based on chi-square test. BMI: body mass index

Variables		Yes		No		X^2^	p-Value*
		N	%	N	%		
Gender	Male	100	93.5	7	6.5	2.357	0.125
	Female	104	87.4	15	12.6		
Age	<18 years	6	75.0	2	25.0	6.148	0.105
	18–35 years	110	88.0	15	12.0		
	36–55 years	84	95.5	4	4.5		
	>55 years	4	80.0	1	20.0		
BMI categories	Within normal	27	96.4	1	3.6	2.667	0.446
	Overweight	65	89.0	8	11.0		
	Obesity	61	92.4	5	7.6		
	Morbid obesity	51	86.4	8	13.6		

## Discussion

According to the data analysis, the distribution was almost equal for males and females. Furthermore, more than half of the participants were 18-35 years old, possibly related to many contributing factors affecting this age group. For instance, people at a young age have a higher tendency to gain more weight, eat unhealthy food, and follow unhealthy habits [3]. Furthermore, many young-aged individuals are trying to lose weight because this age group is concerned about body weight, which could affect employment, marriage, or body image. This viewpoint could explain why this young-aged group is trying to follow new methods and trends of weight-loss diets, including ketogenic diets [8,9].

Moreover, most of the respondents were either overweight, obese, or morbidly obese, and only 12.4% had a normal body mass index. Thus, most of these participants adopted a ketogenic diet to lose weight. A ketogenic diet could be healthy a lifestyle diet, but it is difficult to adopt, as evidenced by the duration of diet commitment. Most of the participants (69.9%) used this diet for more than one month, with 12.8% for six to 12 months and only 7.5% for more than a year. In addition, two-thirds of the participants used this diet once, 14.6% twice, and 17.3% thrice. Therefore, a ketogenic diet is hard to adopt as a lifestyle and maybe only a temporary effective method for weight reduction. Further research is needed to assess its difficulty in the long-term application; perhaps, it may be explained by its side effects or its strict nature.

Overweight individuals can lose more weight through caloric restriction than through exercise, although more of the weight loss by dieting is from lean body mass. However, diet intervention with exercise can lose more weight than diet intervention alone [10]. Accordingly, more than half of our participants performed physical exercise while on a ketogenic diet, whereas 41.6% did not perform any physical exercise. This finding may explain the outcomes of a ketogenic diet, prompting healthcare providers to conduct further education and awareness to the public about the importance of physical activities [11]. Additionally, slightly more than half of the participants (54.4%) had cheat meals, possibly affecting the ketogenic diet outcomes; once they have cheat meals, they are out of the diet. In other words, the exact ketogenic idea is disrupted; thus, the desired results may not rely on whether the participants did not follow the diet again or whether they kept having a weekly cheat day. Moreover, most of the participants based their food preparation on what they learned on social media or simply self-prepared their meals. Only roughly 2% consulted a dietitian or availed of ready-made meals. Individuals aiming for a ketogenic diet should seek the assistance of a dietitian from a scientific perspective to increase awareness and help anyone who wants to try a ketogenic diet in a proper way [12].

Most of the participants (96.9%) lost weight, and approximately 30% showed significant weight loss (≥10 kg). Only five participants did not notice any change, and two gained weight, possibly because of the quality of the diet practice or maybe they are still in the early stage of their diet. The high percentage of weight loss could confirm the efficacy of the ketogenic diet in weight reduction [13].

Weight loss is the probable reason to adopt a diet, and people are more prone to follow any diet with a reputation that goes around weight loss without focusing on the possible side effects or the possible negative health impact. The respondents reported different symptoms and side effects. Frequent side effects were dizziness, nausea, polyurea, halitosis, palpitations, sluggishness, constipation, and muscle pain-all of which have different intensities-either mild, moderate, or severe.

In the first month of adopting a ketogenic diet, the overall self-reported perception of the respondents about their physical and mental health was very positive, excluding the first few days wherein the respondents felt poor physical and mental health that prevented them from doing usual daily tasks for an average of one day. Poor physical and mental health in the first few days of a ketogenic diet can be explained by the body’s effect of switching the use of glucose as the main source of energy to ketone bodies. Generally, most of the respondents (90.3%) felt happy about adopting a ketogenic diet, and 81.9% would recommend this diet for anyone who wants to lose weight.

The study has some limitations. First, it was distributed over the social media group with a special interest in a ketogenic diet, thereby possibly exaggerating the positive attitude toward this diet. Second, the symptoms were derived from previous studies that included the pediatric population, which could have outcomes different from those of the adult population. Finally, this study asked retrospective questions, which could be affected by the whole participant experience and recalling of the symptoms.

## Conclusions

Most of the participants adopted a ketogenic diet for one to six months, indicating that this diet is only a short-term solution to weight reduction. Almost all of the participants lost weight, indicating the efficacy of the ketogenic diet in weight reduction. Different symptoms and side effects were also reported, with varying intensities, especially in the first few days of adopting this diet. Moreover, most of the respondents felt happy about adopting a ketogenic diet and would recommend it to anyone who wants to lose weight. In conclusion, the ketogenic diet did not affect the quality of life and yielded a very positive experience overall.

## Appendices

The ketogenic diet (keto diet)

Disclaimer: This survey is confidential. All data collected from you will be securely stored throughout the duration of the project.

We are a group of medical students at the University of Jeddah. We would like you to participate in the following questionnaire, through which we try to discuss the quality of life as well as the benefits and side effects of the ketogenic diet, as well as for those who do not practice the diet in an attempt to form an integrated idea.

The survey will not take more than two to three minutes of your time. If you have any questions, you can contact us at the following email:

- Please note that once you have answered the questionnaire, it means that you have understood the above and have given your consent to share your data with the researcher. *

o Agree

o Disagree

- Have you tried the ketogenic diet? *

o Yes

o No

Section 1 - Tried Ketogenic Diet

- Age *

o Under 18 years

o 35-18 years

o 36-55 years

o 56-70 years

o Over 70 years

- Sex *

o Male

o Female

Height (centimeter): …………………

Weight (kg): …………………

- How long have you been on the keto diet? *

o Less than a month

o 1-3 months

o 3-6 months

o 6-12 months

o More than 12 months

- Did your commitment to the diet include open meals (cheating meals), and if your answer is yes, how often in a week? *

o No, it did not include open meals

o Yes, one open meal per week

o Yes, two open meals a week

o Yes, intermittently

- How many times have you followed the keto diet? *

o Once

o Twice

o Thrice

o Many times

- Did you exercise while you were following the diet? *

o No, I did not exercise

o Yes, I exercised

- How do you prepare your diet meals? *

o By a nutritionist

o By ready meals

o Via social media

o Through books on nutrition

o By an experienced friend

o By myself

o Other (please specify): …………

- How much weight did you lose during the first three months? *

o My weight has not changed

o Lost 1-3 kg

o Lost 3-5 kg

o Lost 5-10 kg

o Lost 10-15 kg

o Lost >15 kg

o I gained weight

- In the first three months of following the diet, did you suffer from nausea? *

o I did not at all

o Mild

o Moderate

o Very severe

o Not sure

- In the first three months of your diet, did you notice an increase in urination? *

o I did not at all

o Mild

o Moderate

o Very severe

o Not sure

- In the first three months of following the diet, did you notice an increase in heart rate (palpitations)? *

o I did not at all

o Mild

o Moderate

o Very severe

o Not sure

- In the first three months of your diet, were you diagnosed with acidity in the blood? *

o No

o Mild

o Moderate

o Very severe

o Not sure

- In the first three months of following the diet, did you suffer from a fever? *

o I did not at all

o Mild

o Moderate

o Very severe

o Not sure

- In the first three months of following the diet, did you suffer from cold or flu symptoms? *

o I did not at all

o Mild

o Moderate

o Very severe

o Not sure

- In the first three months of your diet, did you suffer from muscle pain? *

o I did not at all

o Mild

o Moderate

o Very severe

o Not sure

- In the first three months of your diet, did you suffer from abdominal pain? *

o I did not at all

o Mild

o Moderate

o Very severe

o Not sure

- In the first three months of following the diet, did you suffer from bad breath? *

o I did not at all

o Mild

o Moderate

o Very severe

o Not sure

- In the first three months of your diet, did you experience a drop in sugar? *

o I did not at all

o Mild

o Moderate

o Very severe

o Not sure

- In the first three months of your diet, did you suffer from lethargy? *

o I did not at all

o Mild

o Moderate

o Very severe

o Not sure

- In the first three months of your diet, did you suffer from constipation? *

o I did not at all

o Mild

o Moderate

o Very severe

o Not sure

- In the first three months of your diet, did you experience dizziness or lightheadedness? *

o I did not at all

o Mild

o Moderate

o Very severe

o Not sure

- In the first three months of your diet, did you suffer from vomiting? *

o I did not at all

o Mild

o Moderate

o Very severe

o Not sure

Have you had any other symptoms? Please mention it:

……………………………………………………………………………………………………………………………………………………………………………………………………………………………………………………

During the first month of following the ketogenic diet and considering your physical health, including side effects and physical problems, approximately how many days of that month were you not in a good health condition? 

Write from 1-31 days: ……….

- Do you feel angry with everyone around you? *

o Yes, most of the time

o Yes, sometimes

o No

- Are you happy with your current health on the ketogenic diet? *

o Yes

o No

- Do you have enough means to do physical activity? *

o Yes

o No

- Would you recommend the keto diet to someone who wants to lose weight? *

o Yes

o No

o Not Sure

Section 2 -  No Keto Diet

- Age *

o Under 18 years

o 35-18 years

o 36-55 years

o 56-70 years

o Over 70 years

- Sex *

o Male

o Female

Height (centimeter): …………………

Weight (kg): …………………

- In the past three months, have you experienced nausea? *

o I did not at all

o Mild

o Moderate

o Very severe

o Not sure

- In the past three months, have you noticed an increase in urination? *

o I did not at all

o Mild

o Moderate

o Very severe

o Not sure

- In the past three months, have you been diagnosed with acidity in the blood? *

o No

o Mild

o Moderate

o Very severe

o Not sure

- In the past three months, have you experienced fever? *

o I did not at all

o Mild

o Moderate

o Very severe

o Not sure

- In the past three months, have you suffered from cold or flu symptoms? *

o I did not at all

o Mild

o Moderate

o Very severe

o Not sure

- In the past three months, have you suffered from abdominal pain? *

o I did not at all

o Mild

o Moderate

o Very severe

o Not sure

- In the past three months, have you experienced muscle pain? *

o I did not at all

o Mild

o Moderate

o Very severe

o Not sure

- In the past three months, have you experienced bad breath? *

o I did not at all

o Mild

o Moderate

o Very severe

o Not sure

- In the past three months, have you experienced low blood sugar? *

o I did not at all

o Mild

o Moderate

o Very severe

o Not sure

- In the past three months, have you suffered from lethargy? *

o I did not at all

o Mild

o Moderate

o Very severe

o Not sure

- In the past three months, have you suffered from constipation? *

o I did not at all

o Mild

o Moderate

o Very severe

o Not sure

- In the past three months, have you experienced dizziness or lightheadedness? *

o I did not at all

o Mild

o Moderate

o Very severe

o Not sure

- In the past three months, have you experienced an increased heart rate? *

o I did not at all

o Mild

o Moderate

o Very severe

o Not sure

- In the past three months, have you experienced vomiting? *

o I did not at all

o Mild

o Moderate

o Very severe

o Not sure

- Did you suffer from other symptoms? Please mention it:

………………………………………………………………………………………………………………………………………………………………………………………………………………………………
